# Raltegravir Is a Potent Inhibitor of XMRV, a Virus Implicated in Prostate Cancer and Chronic Fatigue Syndrome

**DOI:** 10.1371/journal.pone.0009948

**Published:** 2010-04-01

**Authors:** Ila R. Singh, John E. Gorzynski, Daria Drobysheva, Leda Bassit, Raymond F. Schinazi

**Affiliations:** 1 Department of Pathology, University of Utah, Salt Lake City, Utah, United States of America; 2 Center for AIDS Research, Laboratory of Biochemical Pharmacology, Department of Pediatrics, Emory University School of Medicine and Veterans Affairs Medical Center, Decatur, Georgia, United States of America; Institut Pasteur Korea, Republic of Korea

## Abstract

**Background:**

Xenotropic murine leukemia-related retrovirus (XMRV) is a recently discovered retrovirus that has been linked to human prostate cancer and chronic fatigue syndrome (CFS). Both diseases affect a large fraction of the world population, with prostate cancer affecting one in six men, and CFS affecting an estimated 0.4 to 1% of the population.

**Principal Findings:**

Forty-five compounds, including twenty-eight drugs approved for use in humans, were evaluated against XMRV replication *in vitro*. We found that the retroviral integrase inhibitor, raltegravir, was potent and selective against XMRV at submicromolar concentrations, in MCF-7 and LNCaP cells, a breast cancer and prostate cancer cell line, respectively. Another integrase inhibitor, L-000870812, and two nucleoside reverse transcriptase inhibitors, zidovudine (ZDV), and tenofovir disoproxil fumarate (TDF) also inhibited XMRV replication. When combined, these drugs displayed mostly synergistic effects against this virus, suggesting that combination therapy may delay or prevent the selection of resistant viruses.

**Conclusions:**

If XMRV proves to be a causal factor in prostate cancer or CFS, these discoveries may allow for rational design of clinical trials.

## Introduction

Xenotropic murine leukemia-related retrovirus (XMRV) is a recently discovered infectious agent [1] that has been linked to human prostate cancer [2] and chronic fatigue syndrome (CFS) [3]. Both diseases affect a large fraction of the world population, with prostate cancer affecting one in six men, and CFS affecting an estimated 0.4 to 1% of the population [4], [5]. XMRV nucleic acid or proteins are found in 27% of prostate cancers and in 68% of chronic fatigue syndrome patients, and in less than 4–6% of normal controls, suggesting an association between the virus and human disease [2], [3].

CFS, a disease characterized by severe debilitating fatigue, has had an uncertain etiology since its recognition. While a series of viral agents and environmental toxins have been proposed to be associated with CFS, no clear evidence for these has ever been presented (reviewed in [6]). The recent association of XMRV with CFS from the Whittemore Peterson Institute in Reno, Nevada, while far from being proven causal, is the strongest viral association to be made yet. Three recent reports, using plasmid DNA as positive controls, did not find XMRV in CFS patients in Europe [7] [8] [9]. The prevalence of XMRV in prostate cancer in Europe is uncertain, with one German group reporting the presence of XMRV in human prostates [10], and the other not detecting any [11]. However, the notion that a retrovirus might be involved in both cancer and a neuroimmune illness in humans is not without precedence. Human T-cell lymphotrophic virus, type 1 (HTLV-1), another retrovirus, causes both T-cell lymphoma/leukemia as well as tropical spastic paraparesis, a myelopathy due to immune defects resulting from the viral infection.

Infectious XMRV has been isolated from sera of CFS patients [3]. The presence of circulating infectious retrovirus particles in the blood invokes a scenario not unlike infection with another retrovirus, human immunodeficiency virus type 1 (HIV-1). Since there is no effective treatment for this profoundly debilitating illness, the use of antiretroviral agents that have proven to be reasonably safe for human use, might be of benefit. The discovery of effective antiretroviral agents against XMRV would allow for rational design of clinical trials to prevent progression of prostate cancer or to treat CFS.

In this study, we report the effect of 45 compounds on XMRV replication in MCF-7 and LNCaP cells, cell lines generated from human breast and prostate cancers, respectively. We studied drugs used in the treatment of HIV-1 infections, as well as compounds used to treat other viral infections in humans. XMRV is a gammaretrovirus, closely related to the murine leukemia viruses (MLV) [1]. At the amino acid level, it shares considerable identity with sequences of Moloney murine leukemia virus (MoMLV), a prototype MLV. The maximum similarity between XMRV and MoMLV proteins is found in the sequences for viral protease (96% identity), and the least similarity is between the two envelope proteins (66% identity) [1]. Unfortunately, not many actively-used antiretroviral agents have been tested for activity against MLV, with the exception of ZDV, which effectively suppresses MLV [12], and was recently demonstrated to be effective against XMRV as well [13]. In contrast, while there is a lot of information on antiviral activity against essential HIV-1 proteins, there is very little similarity between HIV-1 and XMRV proteins, with the proteases (PR) of the two viruses sharing 28% identity at the amino acid level, the reverse transcriptase proteins (RT) sharing 17% and the integrase (IN) proteins sharing just 14% identity. This low sequence similarity makes it difficult to predict which, if any, of the antiretroviral agents that are effective against HIV-1 would be effective against XMRV. We chose several drugs from each major class of antiretroviral agents: nucleoside and non-nucleoside RT inhibitors (NRTIs and NNRTIs), IN inhibitors, and PR inhibitors (PI). The envelope proteins of the XMRV and HIV-1 are widely divergent in size (70 kD and 160 kD respectively), utilize different receptors for viral entry and do not share any significant similarity. Therefore, peptidomimetics that act on the HIV-1 envelope protein to prevent viral entry were not included in our study. A few inhibitors that are known to inhibit replication of viruses other than retroviruses were also evaluated. A significant number of compounds tested in our study, viz. 28 out of a total of 45, are already FDA-approved for the treatment of infection with HIV-1 or other viruses. We report here for the first time that the integrase inhibitor, raltegravir (RAL), is extremely potent and selective against XMRV, when used at low submicromolar concentrations in both cell culture systems. Another IN inhibitor, L-000870812, and two NRTIs, ZDV and tenofovir disoproxil fumarate (TDF), also inhibit XMRV replication, but at higher concentrations. When combined, these compounds display synergistic effects, suggesting combined modalities to treat XMRV infection, thus delaying or preventing the selection of resistant viruses.

## Results

**Figure 1:**
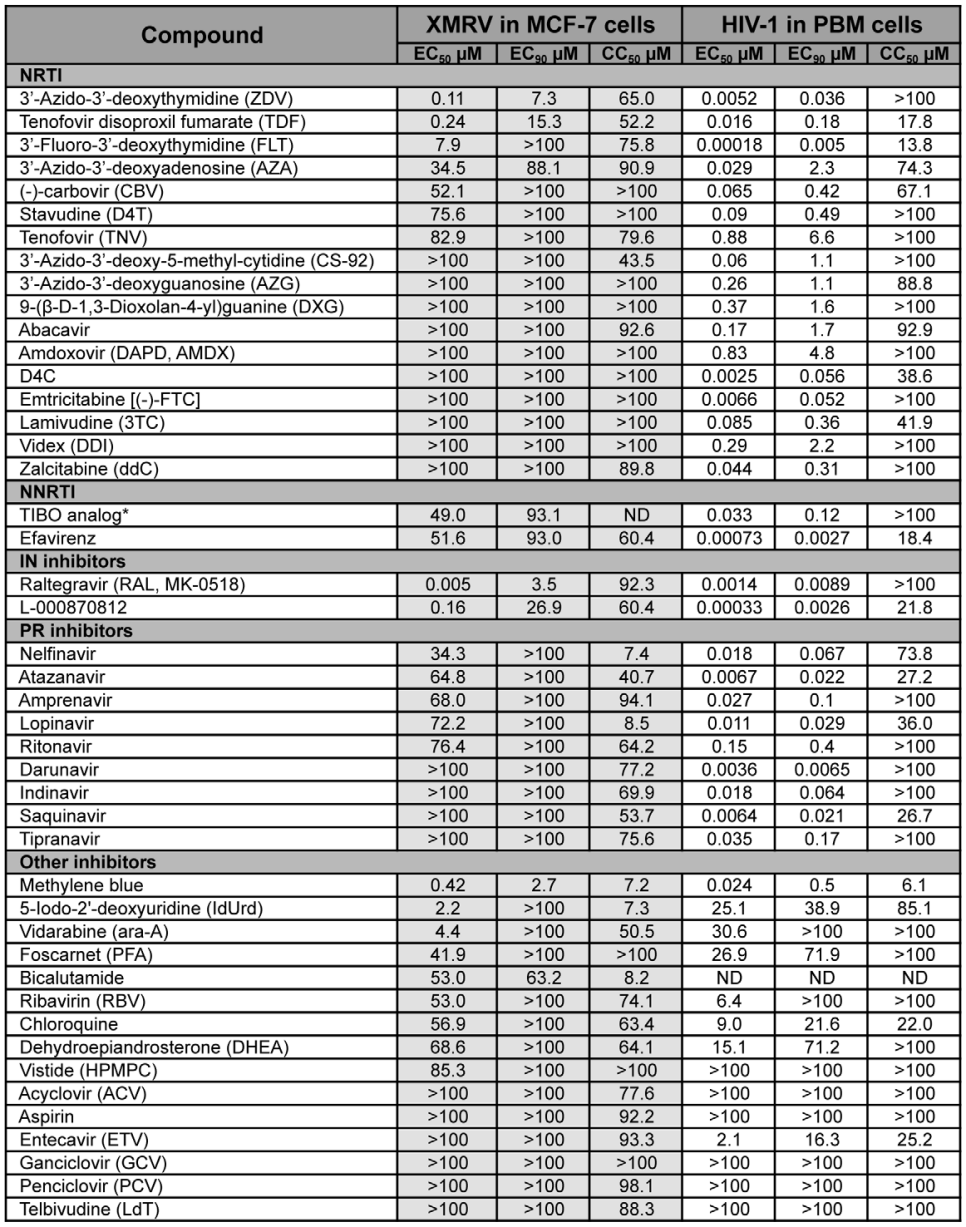
EC_50_, EC_90_ and CC_50_ values of compounds tested in XMRV-infected MCF-7 cells, and in HIV-1 infected peripheral blood mononuclear cells. All compounds were evaluated in duplicate at least three times. Values shown are average of replicate assays. *(4*S*)-8-Chloro-4-methyl-5-(3-methylbut-2-enyl)-3,4,5,6-tetrahydro-1H-[1], [3]diazepino[4,5,6-cd]indole-2(2aH)-thione.

**Figure 2:**
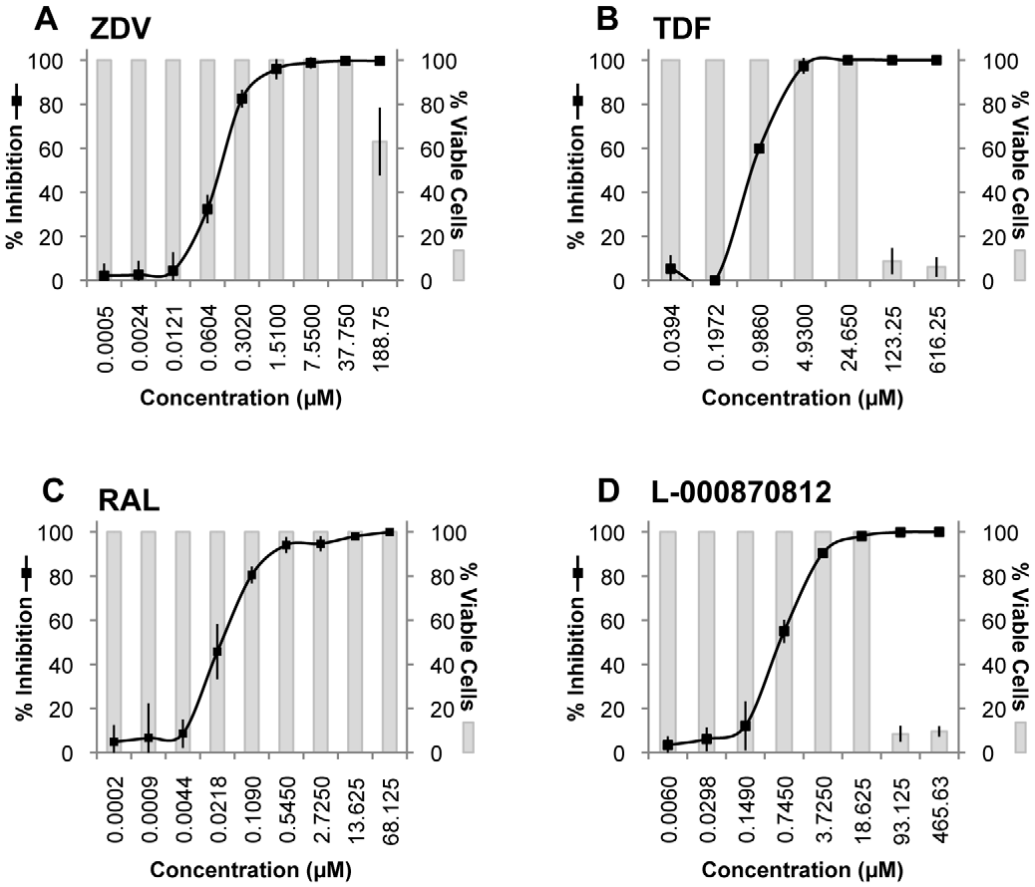
Inhibition of XMRV replication in LNCaP cells in the presence of increasing concentrations of antiviral agents. Viral release from XMRV-infected LNCaP cells in the presence of increasing concentrations of (A) ZDV (B) TDF (C) RAL and (D) L-000870812, was determined by measuring RT activity in the supernatants. Percent inhibition was calculated based on infected cells exposed to DMSO alone being set to 0% inhibition, and naïve cells in the absence of any compounds set at 100% inhibition. Cell viability was checked by microscopy, quantified by the MTT assay, and represented by shaded bars. Data for each compound were derived from an average of at least three independent experiments, each performed in duplicate.

We tested a total of 45 compounds belonging to different classes of HIV-1 inhibitors, and a few inhibitors of viruses other than retroviruses, for their ability to inhibit XMRV replication in cultured cells. LNCaP and MCF-7 cells were chosen for their ability to support robust *in vitro* replication of XMRV. MCF-7 cells, because of their better growth properties in culture were initially used to test all 45 compounds (Figure 1). Compounds with anti-XMRV activity were subsequently tested in both LNCaP and MCF-7 cells (Table S1). To determine if a reduction in viral release might be due to toxicity of the compound and not due to specific antiretroviral activity, cellular morphology was monitored every 24 h by microscopic examination, and an MTT [3-(4,5-dimethylthiazol-2-yl)-2,5-diphenyltetrazolium bromide] colorimetric assay was used to measure potential cytotoxicity produced by the compounds. Supernatants were collected every 24 h and assayed for viral release by measuring RT activity. Inhibition of RT activity (see Figure 2) was averaged over 3–6 experiments, each performed in duplicate, and used to calculate the median (EC_50_) and 90% effective concentrations (EC_90_) for each compound (Figure 1). For comparison purposes, all compounds evaluated in these studies were also tested against HIV-1_LAI_ in primary human lymphocytes.

### Inhibitors of HIV-1 reverse transcriptase

The following NRTI inhibitors of HIV-1 RT were tested in our XMRV replication assays: ZDV, 3′-azido-3′-deoxyadenosine (AZA), 3′-azido-3′-deoxyguanosine (AZG), 3′-azido-3′-deoxy-5-methyl-cytidine (CS-92), lamivudine (3TC), emtricitabine [(-)-FTC], tenofovir (TNV) and its prodrug form TDF, 9-(β-D-1,3-dioxolan-4-yl)guanine (DXG) and its prodrug form, amdoxovir (DAPD, AMDX), (-)-carbovir (CBV), stavudine (D4T) and its corresponding cytosine analog (D4C), videx (ddI), zalcitabine (ddC), and 3′-fluoro-3′-deoxythymidine (FLT). We also tested the NNRTIs efavirenz, and a TIBO derivative that was shown to be effective in a murine system [14]. Among these, the most potent XMRV inhibitors were ZDV and TDF (Figure 2, A, B). The EC_50_ and EC_90_ in MCF-7 cells were 0.11 µM and 7.3 µM for ZDV, and 0.24 µM and 15.3 µM for TDF respectively (see Figure 1). The EC_50_ and EC_90_ values were also determined in LNCaP cells, a prostate cancer cell line, and there was a consistent difference of up to 5 fold between the two cell lines, which may be related to their differing rates of nucleoside uptake and bioconversion to the active nucleoside triphosphate analog. The EC_50_ and EC_90_ in LNCaP cells were 0.14 µM and 1.1 µM for ZDV, and 0.9 µM and 4.2 µM for TDF, respectively. CBV, AZA, FLT and D4T all showed greater than 70% inhibition of XMRV replication (see Table S1), but at the much higher concentration of 100 µM. AZG, CS-92, (-)-FTC, 3TC, ddI, DAPD and DXG were essentially inactive at 100 µM (Figure 1). TFV was also ineffective against XMRV, probably due to its polar nature, which may not allow sufficient drug to penetrate into the cells. The NNRTIs efavirenz (EFV) and the TIBO derivative, did not demonstrate any major activity against XMRV.

### Inhibitors of HIV-1 Integrase

IN inhibitors raltegravir (RAL or MK-0518) and L-000870812 [15] were also evaluated for their ability to inhibit XMRV in the two cell systems (Figure 1, Figure 2 C and D). Of all the compounds tested, RAL was the most potent, with an EC_50_ of 0.005 µM and an EC_90_ of 3.5 µM in MCF-7 cells, and an EC_50_ of 0.03 µM and an EC_90_ of 0.46 µM in LNCaP cells (Table S1). L-000870812, showed activity against XMRV replication at considerably higher concentrations, with an EC_50_ and EC_90_ of 0.16 µM and 26.9 µM in MCF-7 cells, and 0.7 µM and 4.5 µM in LNCaP cells, respectively.

### Inhibitors of HIV-1 Protease

Nine known HIV-1 PIs were evaluated for activity against XMRV (Figure 1). The most effective was nelfinavir, albeit with an EC_50_ of 34.3 µM. The following PIs had very modest anti-XMRV activities: atazanavir (EC_50_ of 64.8 µM), amprenavir (EC_50_ of 68.0 µM), lopinavir (EC_50_ of 72.2 µM), and ritonavir (EC_50_ of 76.4 µM). Darunavir, indinavir, saquinavir and tipranavir were essentially ineffective against XMRV *in vitro*, when tested up to 100 µM.

### Inhibitors of viruses other than HIV-1

A select number of antiviral agents known to inhibit viruses other than retroviruses were also evaluated. These included the anti-herpetic drugs acyclovir (ACV), ganciclovir (GCV), vidarabine (ara-A), 5-Iodo-2′-deoxyuridine (IdUrd), penciclovir (PCV), foscarnet (PFA), vistide (HPMPC); the anti-hepatitis drugs entecavir (ETV), telbivudine (LdT), and ribavirin (RBV). ETV was also selected because it was recently reported to inhibit HIV-1 replication, both *in vitro* and in humans [16]. Other compounds claimed to be effective against XMRV, MLV, HIV-1 and other viruses, such as chloroquine [17], dehydroepiandrosterone (DHEA) [18], methylene blue and aspirin were also evaluated for anti-XMRV activity *in vitro*. Methylene blue is known to have antiherpetic activity and also can inactivate HIV-1 [19]. Unfortunately, most of the compounds listed above, except IdUrd were ineffective against XMRV, or were effective at toxic concentrations (Figure 1). IdUrd demonstrated a low therapeutic index (TI, the ratio of CC_50_/EC_50_) and cannot be considered as a specific antiviral agent against XMRV.

### Combination effects of active compounds on XMRV replication

**Figure 3:**
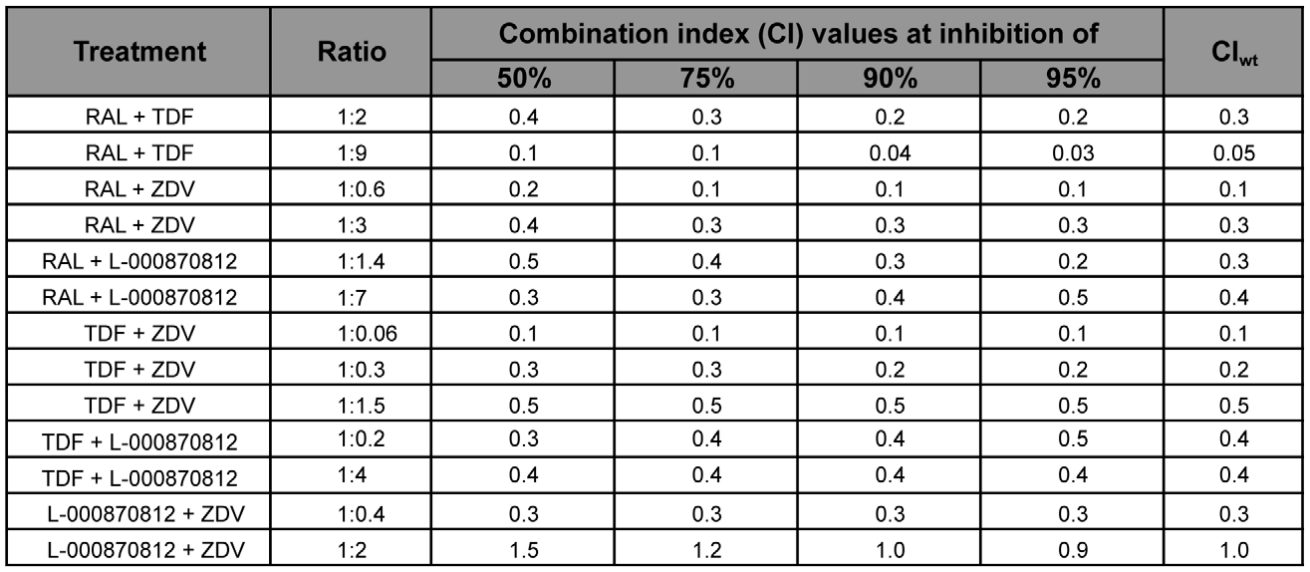
Evaluation of drug-drug interactions against XMRV at 50%, 75%, 90%, and 95% inhibition. Combination Index (CI) values were determined for a mutually exclusive interaction using CalcuSyn program, where CI <1 indicates synergism, CI = 1 indicates additive effect, and CI >1 indicates antagonism. Weighted average CI value (CI_wt_) was assigned as [CI_50_ + 2CI_75_ + 3CI_90_ + 4CI_95_]/10. RAL, raltegravir; TDF, tenofovir disoproxil fumarate; ZDV, zidovudine.

Binary combinations of the most potent compounds, viz. RAL, L-000870812, TDF and ZDV were tested for activity against XMRV in LNCaP cells. Compounds were tested at increasing concentrations, in three different sets, with the ratio of the two compounds kept constant. The data were analyzed using the CalcuSyn method originally described by Chou and Talalay [20]. A summary of results for all combinations evaluated in LNCaP cells is presented in Figure 3.

For the combinations tested (RAL with TDF or ZDV or L-000870812; TDF with ZDV or L-000870812; L-000870812 with ZDV), an additive or mostly synergistic interaction was noted at all effect levels without apparent cytotoxicity at the highest concentrations used. In the computational analysis for either one of the four drugs (RAL, TDF, L-000870812, or ZDV), the linear correlation coefficient (*r* values) of the median-effect plot or for their constant ratio combinations ranged from 0.92 to 0.99 (data not shown), matching the law of mass-action. The *in vitro* effect of the combination of RAL with either TDF or ZDV, showed a favorable dose reduction at all ratios. In addition, all Combination Index (CI) values (see Materials and Methods) were less than 1, suggesting synergy when the combination ratios of either TDF or ZDV and RAL were analyzed (Figure 3). In addition, dual combinations of either ZDV and L-000870812 or TDF were also tested. The weighted CI (CI_wt_) values of 0.1 to 0.5 for TDF + ZDV, indicated synergistic effects at all ratios tested (Figure 3). Moreover, the dual combination of L-000870812 and ZDV at ratio 1∶2 indicated a nearly additive effect (CI_wt_ of 1.0); however at ratio of 1∶0.4, the CI value was 0.3, indicating synergism (Figure 3). Of significance was that all dual combinations containing RAL, the most potent antiviral agent against XMRV, demonstrated marked synergy at all effect levels without any apparent cytotoxicity. Interestingly, the combination of the two IN inhibitors was not antagonistic or additive, but was found to be synergistic, suggesting that these two compounds may have different antiviral mechanisms (see discussion).

## Discussion

In the absence of a clear etiology, the treatment of CFS has been both empirical and unconventional. Therapies have included immunostimulant therapy through injections of staphylococcus toxoid [21], intravenous immunoglobulin therapy [22] [23] [24], and hydrocortisone [25] each with uneven results. Interferon-β and TNF-α inhibitors have been tried in very small numbers of patients. Anti-depressants, NSAIDs, anxiolytic drugs, stimulants, anti-allergy drugs and anti-hypotensive drugs have all been used, but are not universally beneficial [26]. The lack of effective therapy has led to use of plant extracts [27], homeopathy [28], [29], hypnosis [30], acupuncture [31], and whole body periodic acceleration stress [32], none with sustained benefits. The only modalities of treatment that have any proven benefits are cognitive behavioral therapy and graded exercise programs, both of which appear to aid by improving coping skills rather than reduce symptoms [33]. If, XMRV proves to have a causal association with human disease, then the knowledge that certain antiretroviral agents inhibit XMRV at submicromolar concentrations *in vitro*, and have synergistic effects when combined, as shown in this study, might lead to clinical trials. We found that RAL, L-000870812, ZDV, and TDF strongly inhibit XMRV in cell culture, with RAL being the most potent, at an EC_50_ of 0.005 µM, and others such as L-000870812 (EC_50_ = 0.16 µM), ZDV (EC_50_ = 0.11 µM) and TDF (EC_50_ = 0.24 µM) being quite effective as well. In addition, these compounds had high therapeutic indices, with values for ZDV = 591; TDF = 218; RAL = 18,460 and L-000870812 = 378, indicating that it should be possible to achieve therapeutic antiviral levels with minimal toxicity.

Several compounds that we evaluated had a limited effect on XMRV replication *in vitro*. Some of these effects can be explained by currently understood mechanisms. For example, both 3TC and (-)-FTC need a functional YMDD motif in RT to be active. The M184V mutation in HIV-1 RT makes the virus resistant to 3TC and (-)-FTC [34]. These drugs are ineffective against MoMLV, because in MoMLV RT, V is the natural residue in this motif in place of M. Similarly, V is also the natural residue at this location in XMRV RT, making 3TC and (-)-FTC ineffective. Why none of the HIV-1 PIs were effective against XMRV (a finding that has been reported for selected PIs before [13]) remains unclear at this time, but could be related to the size of the PI pocket as well as other biochemical and structural factors.

There was a difference in activities of compounds when tested in different cell types, which may be related to drug uptake by cells, the different levels of natural dNTP in the cells, as well as different intracellular phosphorylation capacity [35]. In general, the EC_50_ for the active compounds listed above were lower in MCF-7 than LNCaP cells suggesting greater potency. Relative to HIV-1, the compounds were generally less potent against XMRV than HIV-1, especially at the EC_90_ level.

The use of monotherapy for treating HIV infections has lead to the appearance of drug resistant virus [36], [37]. The finding that RAL, L-000870812, TDF and ZDV have strong synergistic effects when combined in dual combination bodes well for combination therapy in case of XMRV infection. If XMRV infection parallels other retroviral infections, then the use of combination antiretroviral therapy might maintain XMRV suppression, prevent the emergence of resistance to antiretroviral agents and possibly also cause amelioration of disease. For HIV-1, combination therapy works especially well when the combined drugs have different viral protein targets, or in the case of nucleosides, utilize different kinases for their activation to NTP analogs [31]. We, therefore, judiciously selected drug combinations that inhibit XMRV, such as RAL with ZDV or TDF or L-000870812. When the data were analyzed using the robust method of Chou and Talalay, additive or synergistic interactions were found at all effect levels when these agents were tested in LNCaP cells. Of significance was that no antagonism was noted for any of the combinations evaluated in these cells. To our surprise, even the two IN inhibitors displayed a synergistic effect. Both IN inhibitors act by inhibiting the strand transfer reaction, but if their mechanism of action were to be identical, they would display an additive effect in combination. A synergistic effect suggests that there might be subtle mechanistic differences in the actions of these two IN inhibitors, a finding that is corroborated by unpublished biochemical experiments (personal communication, Dr. Daria Hazuda, Merck Research Laboratories, West Point, PA). It is important to note here, that XMRV differs from HIV-1 in one aspect that is significant for these studies: XMRV isolates show very limited sequence diversity compared to HIV-1 or MLV. Of all the sequenced XMRV isolates that currently exist, both from cases with prostate cancer as well as CFS, obtained from geographically distant parts of the United States, the two least related genomes differ from each other in only 27 out of a total of over 8,100 nucleotides. A similar degree of limited genetic diversity has been found for HTLV-1 [38], another retrovirus implicated in both cancer and neuroimmune illness. It has been suggested that this lack of diversity in XMRV sequences implies that the number of replication cycles within one infected individual is limited [39]. This would suggest that XMRV has a considerably lower potential for developing drug-resistant mutations, as compared to HIV-1. Furthermore, it is likely that a combination of just two drugs might be sufficient for preventing the emergence of drug-resistant mutant virus, though this would need to be tested before any therapeutic recommendations can be made. We have attempted to select for RAL resistant viruses in culture for several months now, and have not yet been successful at isolating drug resistant viruses.

When an assay to measure XMRV viral loads becomes available, virus levels in the blood might become an objective surrogate marker for an effective response to antiviral drugs, in addition to clinical outcomes. While it is not yet clear if any illnesses are directly caused by XMRV, our data indicates that XMRV infections might be prevented or treated with specific antiviral agents. In the presence of any evidence of causality of human disease, our findings should provide the basis for designing clinical trials to treat them.

## Materials and Methods

### XMRV, Cells and Infection with XMRV

293T cells (ATCC, CRL-11268) were transfected with pXMRV1, an infectious clone of XMRV [2]. Virus released in the supernatant was harvested and titrated by inoculating MCF-7 cells, a breast cancer cell line (ATCC, HTB-22) at 70% confluence, with a series of ten-fold dilutions of XMRV in serum-free medium, followed 36–48 hrs later by fixation of cells in paraformaldehyde and processing for immunofluorescence using a rabbit antiserum developed against inactivated XMRV [2]. Typical virus preparations gave titers of approximately 2–5×10^6^ infectious units/ml. Virus was diluted in serum-free medium and used to inoculate cells at a multiplicity of infection (MOI) of approximately 3, in the presence of various antiviral compounds as described below. LNCaP, a prostate cancer cell line (ATCC, CRL-1740) and MCF-7 cells were grown to about 50% confluence in DMEM containing 10% heat inactivated fetal bovine serum, 100 µg/ml penicillin, and 100 IU/ml streptomycin. Cells were washed twice with Dulbecco's phosphate buffered saline (DPBS, Gibco), and incubated with the viral inoculum for 90 min at 37°C in the presence of 95% air and 5% CO_2_, cells were washed twice with DPBS, detached with Trypsin-EDTA (0.25% Trypsin; Cellgro), and counted. One thousand cells were added to each well of a 96-well plate, along with an equal volume of medium containing the retroviral inhibitor at 2-fold the desired concentration. Inhibitors were dissolved in DMSO or water, depending on their solubility, and were all tested at 0.01 to 100 µM in 10-fold increments. Where the drug was found to be active at 0.01 µM, further dilutions from 1 nM to 0.01 nM were tested. Each inhibitor was tested in duplicate a minimum of three separate times in MCF-7 cells in a completely blind fashion using coded compounds, and the results averaged. Active compounds were also evaluated for antiviral activity in LNCaP cells to confirm activity in a secondary cell line known to support XMRV replication. For controls, wells containing water or DMSO at appropriate concentrations were used.

### Assays for cytotoxicity and XMRV replication

Each well was carefully monitored for signs of cellular toxicity due to the inhibitors by microscopic observation every 24 h. In addition, cell viability was measured using the CellTiter 96 AQueous One Solution cell proliferation assay according to the manufacturer (Promega, Madison, Wis). Viral release from the cells was assayed by measuring RT activity in the supernatant. For this, supernatant from each well was collected every 24 h and frozen at −20°C until it was analyzed by RT assay for viral release as described previously [40]. In brief, oligo(dT)·poly(rA) primer-template assays were performed in the presence of radiolabeled [α-^32^P]dTTP and Mn^2+^. After incubating the viral supernatants with the RT reaction mix for 1 h at 37°C, samples were spotted onto DE81 DEAE cellulose paper (Whatman) and unincorporated label washed away with 2× SSC (1× SSC = 0.15 M NaCl and 0.015 M sodium citrate). Virion-associated RT was analyzed using a Typhoon 9410 PhosphorImager (GE Healthcare) and quantified with the Image J software (http://rsbweb.nih.gov/ij/). Inhibition of viral release as measured on day 6 after inoculation was averaged over 3–5 experiments and plotted. The antiviral EC_50_ and cytotoxic concentrations (CC_50_,) was determined from the concentration–response curve using the median effect method [20]. HIV-1 replication assays were performed as described previously using peripheral blood mononuclear cells (PBMC) obtained from the American Red Cross, Atlanta, GA, that were stimulated with phytohemagglutinin (PHA) for 72 hr [41].

### Combination studies

To evaluate whether the antiviral effects of dual drug combinations of: RAL with TDF or ZDV or L-000870812; TDF with ZDV or L-000870812; L-000870812 with ZDV were synergistic, additive or antagonistic, drug combinations at several constant ratios were evaluated. RAL, L-000870812, ZDV and TDF were first tested alone to determine the EC_50_ and EC_90_ values, at least three times, each in duplicate. For the median-effect analysis, the compounds were combined at several ratios based on multiples of their EC_50_ or EC_90_ values. For each drug (alone or in combination), three to four independent experiments were performed and all samples were processed in duplicate. Analysis was performed using the software CalcuSyn (Biosoft, Ferguson, MO, USA) (see Figure 3 for CI values), which allows automated simulation of synergism and antagonism at all dose and effect levels and displays the methods of Chou and Talalay [20], including median effect plot and CI values. Because the high degree effects are more therapeutically relevant than the low degree of effects, the additional weighted average CI (CI_wt_) was calculated, which uses the formula: CI_wt_  =  [CI_50_ + 2CI_75_ + 3CI_90_ + 4CI_95_]/10, where CI_50_, CI_75_, CI_90_, CI_95_ are the CI values at 50%, 75%, 90% and 95% inhibition, respectively. [20], [42].

## Supporting Information

Table S1 EC50, EC90 and CC50 values of compounds active against XMRV in MCF-7 cells, as tested in XMRV-infected LNCaP cells. Compounds found to have significant activity in MCF-7 cells were tested in LNCaP cells for activity. All compounds were evaluated in duplicate at least three times. Values shown are average of replicate assays. Corresponding values in MCF-7 cells are shown for comparison.
(0.11 MB TIF)
