# Supplementary figures and images for: Raltegravir Is a Potent Inhibitor of XMRV, a Virus Implicated in Prostate Cancer and Chronic Fatigue Syndrome
Source: PLoS One. 2010 Apr 1;5(4):e9948. doi: 10.1371/journal.pone.0009948 (PMC2848589; doi:10.1371/journal.pone.0009948)

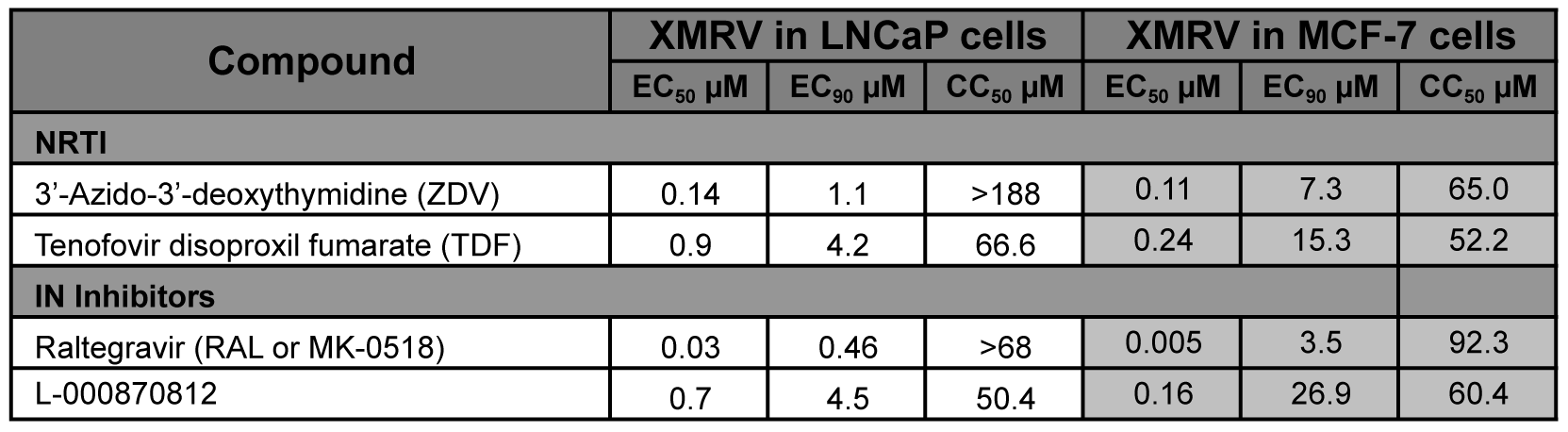

Supplement: Table S1 — EC50, EC90 and CC50 values of compounds active against XMRV in MCF-7 cells, as tested in XMRV-infected LNCaP cells. Compounds found to have significant activity in MCF-7 cells were tested in LNCaP cells for activity. All compounds were evaluated in duplicate at least three times. Values shown are average of replicate assays. Corresponding values in MCF-7 cells are shown for comparison. (0.11 MB TIF) [file pone.0009948.s001.tif]
